# The complete chloroplast genome sequence of *Sedum lineare* (crassulaceae)

**DOI:** 10.1080/23802359.2021.1977196

**Published:** 2021-11-17

**Authors:** Yigong Tang, Haoyu Zhang, Xiaoting Xu, Hong Chang

**Affiliations:** Key Laboratory of Bio-Resource and Eco-Environment of Ministry of Education, College of Life Sciences, Sichuan University, Chengdu, Sichuan, China

**Keywords:** *Sedum lineare*, chloroplast genome, phylogenetic tree

## Abstract

The complete chloroplast genome of *Sedum lineare* Thunberg, a plant widely occurring in most southern provinces of China, is assembled and characterized using Illumina sequencing data in this study. The genome is 149,257 bp in length, containing a large single-copy (LSC) region of 80,963 bp, a short single-copy (SSC) region of 16,648 bp, and two inverted repeat (IR) regions of 25,823 bp. It contains 130 genes, with 85 protein-coding genes, 8 rRNA genes and 37 tRNA genes. Moreover, the phylogenetic tree shows that *S. lineare* is closely related to *Sedum japonicum.*

Known for the special Crassulacean acid metabolism (CAM) photosynthetic pathway, Crassulaceae are a diverse family with approximately 1400 species from ca. 33 genera (Gontcharova and Gontcharov 2009). Of these genera, *Sedum* L., is the most species-rich, morphologically diverse, and taxonomically complex one (Nikulin et al. 2016). *Sedum lineare* Thunberg is a typical perennial herb, which is widely distributed in most southern provinces of China (Rao 1996). In this article, we reported the complete chloroplast genome of *S. lineare*, providing a valuable genomic resource for future studies on genetic diversity of genus *Sedum*.

Fresh leaves of *S. lineare* were collected from Yunnan Province, China (geographic coordinate: 25°8′36.2″ N, 102°44′24.15″ E), and dried with silica gels. The voucher specimen (ZYC190411) was then stored in the Sichuan University Herbarium (SZ). Total genomic DNA was extracted using a modified CTAB method (Doyle and Doyle 1987). The library with an insertion size of ∼300 bp fragments was constructed and sequenced using the Illumina platform. The filtered reads were assembled through the NOVOPlasty v2.7.2 software (Dierckxsens et al. 2017), with the sequenced chloroplast genome *Sedum sarmentosum* (NC_023085) as reference. The assembled chloroplast genome annotation was performed using Plann v1.1 (Huang and Cronk 2015) and the genome was manually inspected using Geneious v11.0.3 (Kearse et al. 2012). The complete chloroplast genome with its gene annotations of *S. lineare* was deposited in GenBank (Sayers et al. 2020) under the accession number MT755626. To further clarify the phylogenetic position of the species, plastomes of five additional Crassulaceae species and one Penthoraceae species were obtained from NCBI to construct a maximum likelihood tree. All the sequences from their 7 chloroplast genomes were aligned with MAFFT v1.3.13 (Katoh and Standley 2013), then a Maximum-likelihood (ML) based phylogenetic tree was constructed using RAxML v8.2.11 (Stamatakis 2014) with 1000 rapid bootstrap replicates (Stamatakis et al. 2008). The GTRGAMMA model was used in the ML analysis.

The chloroplast genome of *S. lineare* is 149,257 bp in length, with a large single copy (LSC) of 80,963 bp, a short single copy (SSC) of 16,648 bp, and two inverted repeat regions (IR) of 25,823 bp for each. The overall GC-content of the chloroplast genome is 37.9%, while the corresponding values of the LSC, SSC, and IRs regions are 35.9%, 31.8%, and 43.0%, respectively. Moreover, the chloroplast genome possesses 130 genes in total, including 85 protein-coding genes, 8 rRNA genes and 37 tRNA genes. The phylogenetic tree shows that the species is closely related to *S. japonicum* with a 100% bootstrap support (Figure 1). The published *S. lineare* chloroplast genome provides useful genetic information for further study on phylogeny and evolution of Crassulaceae.

**Figure 1.**
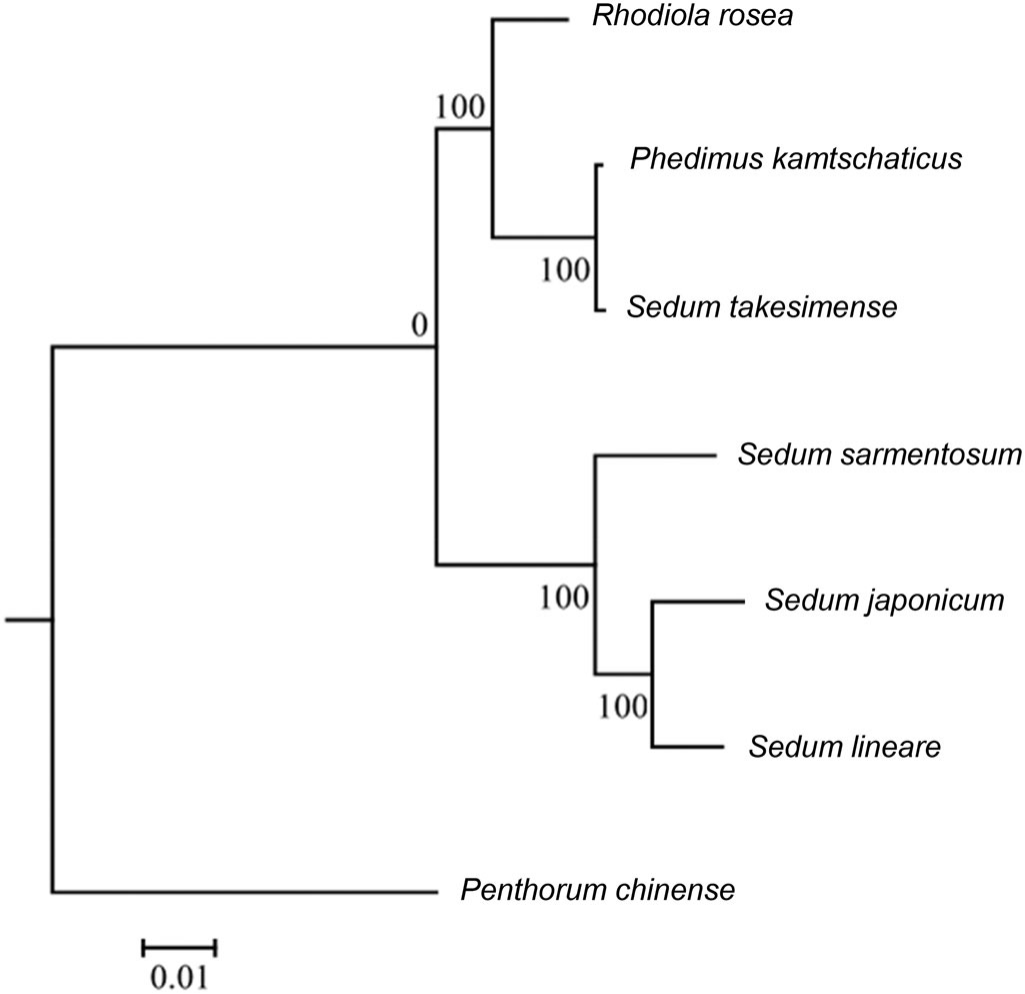
Phylogenetic tree of 7 species based on complete chloroplast genome sequences. Bootstrap percentages are indicated for each branch respectively.

## Data Availability

The data that support the finding of this study are open available in NCBI at http://www.ncbi.nlm.nih.gov/, reference number [MT755626], or available from the corresponding author.
